# Alcohol and Other Drug Abuse Among Women

**Published:** 1994

**Authors:** Barbara W. Lex

**Affiliations:** Barbara W. Lex, Ph.D., M.P.H., is an associate professor in the Department of Psychiatry, Harvard Medical School, Boston, and in the Alcohol and Drug Abuse Research Center of McLean Hospital, Belmont, Massachusetts

## Abstract

Although overall, greater numbers of men than women tend to abuse alcohol and other drugs, equal proportions of both genders in treatment for substance abuse have used multiple substances. Women’s patterns and consequences of alcohol and other drug abuse appear to be influenced by factors that differ from those of men.

The patterns, consequences, and reasons that women abuse alcohol and other drugs differ from those of men. This article reviews such male-female disparities by focusing on selected studies of alcohol, marijuana, cocaine, and opiate use among women. Given existing variations in the research methods used in these studies, it is necessary to acknowledge certain caveats when interpreting the findings. For example, definitions of terms such as “heavy use” may shift over time; definitions of terms such as “alcohol abuse,” “alcoholism,” “drug use,” and “drug abuse” may differ from study to study.^1^ In addition, although men and women may exhibit different profiles of alcohol and other drug use, studies often do not include issues pertinent to women, such as effects of these substances on the menstrual cycle or on pregnancy, when they address issues important for men. More regrettably, in some cases, studies may include data that are obtained from both women and men, but the results are not differentiated by gender (Mello 1983; Mello et al. 1989; Lex 1987).

## The Scope of Alcohol and Other Drug Abuse

**Figure 1:**
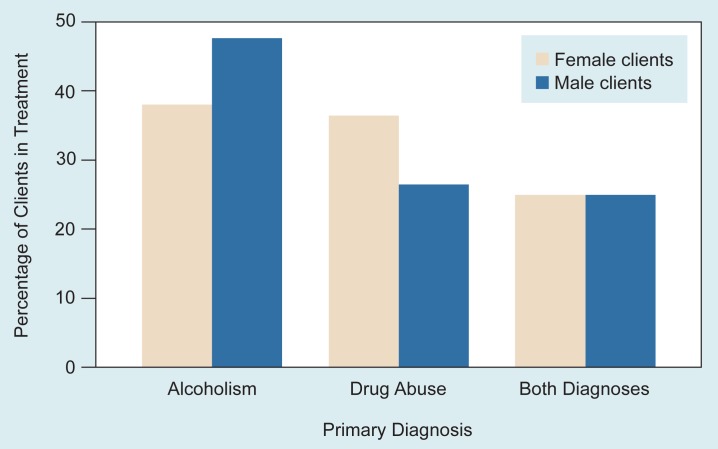
Comparison of male and female clients in treatment for abuse of alcohol and other drugs. Higher proportions of males were in treatment for alcoholism, whereas higher proportions of females were in treatment for drug abuse. Equal proportions of males and females were in treatment for abuse of both alcohol and other drugs. SOURCE: Substance Abuse and Mental Health Services Administration 1993.

In general, men consume more alcohol or use illicit (i.e., illegal) drugs more than do women. However, information about people in treatment for abuse of alcohol and other drugs has shown that although proportionately more men abuse alcohol, a higher proportion of women abuse other drugs (figure 1). In a survey of treatment facilities, alcoholism was diagnosed in fewer women (38.2 percent) than men (47.9 percent); other drug problems were diagnosed in more women (36.4 percent) than men (26.7 percent); and both alcohol and other drug problems were diagnosed with the same frequency in women and men (25 percent) (Substance Abuse and Mental Health Services Administration [SAMHSA] 1993). Although it might have been predicted that more men than women would have alcohol problems, the finding that equal proportions of women and men have sought treatment for concurrent problems with alcohol and illicit drugs was unexpected. The higher proportion of women than men in treatment for illicit drug abuse also was surprising.

Women in treatment for alcoholism frequently have been found to abuse one or more other drugs as well. Compared with their nonalcoholic peers, alcoholic women in one study (Gomberg 1989) reported having used more cocaine (29 percent versus 16 percent), heroin (8 percent versus 1.5 percent), or marijuana (53 percent versus 50 percent). The youngest alcoholic women (ages 20 to 29) typically reported using combinations of alcohol and illicit drugs, whereas the older alcoholic women (in their late thirties and forties) were more likely to use alcohol along with medications, mainly tranquilizers, prescribed by a physician. Accordingly, issues of multiple substance abuse should be considered when planning treatment facilities to meet women’s needs and when obtaining substance abuse histories from women seeking help for abuse problems.

## Survey Data of Alcohol and Other Drug Use

### Household Survey Data

The national household surveys, initially conducted by the National Institute on Drug Abuse (NIDA) and now by SAMHSA’s Office of Applied Studies, are conducted at 1- to 3-year intervals and are intended to provide estimates of the prevalence of alcohol, tobacco, and illicit drug use among the civilian U.S. population ages 12 and older. The 1992 survey interviewed almost 29,000 persons in groups of 118 sampling units (geographically based areas that include households, college dormitories, and homeless shelters) but did not interview transient populations such as homeless people not living in shelters.

#### Alcohol Use

Among the vast majority of survey respondents over age 12 who had used alcohol at least once during their lifetime, slightly more were male (NIDA 1993) (table 1). This pattern also was true for the approximately two-thirds of respondents who had used alcohol during the previous year and the one-half who had used it during the previous month. Rates for males and females over age 18 who had consumed alcohol during the past year were most similar in the age group of 18 to 25.

For those respondents in the age group 12 to 17, the period during which most alcohol users begin consumption, almost 40 percent had ever tried alcohol, and rates for males and females tended to be similar. Although some variation occurs because of sampling error,^2^ about 5 percent more males than females had ever tried alcohol, whereas rates of use by males and females within the past year and within the past month differed about 2 percent to 3 percent. This pattern also was true for the approximately two-thirds of respondents who had used alcohol during the previous year and the one-half who had used it during the previous month (table 1).

#### Illicit Drug Use

Respondents’ use of any illicit drug was assessed to give an overall idea of the prevalence of this activity in the U.S. population. Specific questions about marijuana, cocaine, and heroin use also were asked to estimate a comparison of these drugs’ prevalence to each other as well as to assess multiple substance use in the population. In general, more males than females acknowledged that they had ever used an illicit drug, such as marijuana or cocaine (31.7 percent of the females and 41.0 percent of the males). Only 11.1 percent of those surveyed acknowledged illicit drug use within the past year and 5.5 percent indicated use during the past month (table 1) (NIDA 1993).

Among adolescents ages 12 to 17, however, females were found to have slightly higher rates of illicit drug use than males in each category of use. The higher rates for females were surprising and counterintuitive (given that males typically consume more alcohol and other drugs than females) and perhaps reflected the typical pattern of younger girls dating older boys (Ferrence and Whitehead 1980; Lex 1991*a*). The likelihood of sampling error increases with small percentages, but about 0.3 percent fewer males than females reported that they had ever tried an illicit drug (table 1).

When comparing use of alcohol and other drugs by males and females, a general pattern of prevalence arises. Although men reported somewhat higher overall use of each type of drug, rates for men and women over age 18 for use of alcohol and other drugs were most similar among people ages 18 to 25. Rates of use across substances also were similar among adolescent males and females. Male and female rates for marijuana, cocaine, and heroin use agreed generally with this pattern, but each had unique characteristics that are discussed below (NIDA 1993).

#### Marijuana Use

In general, using marijuana at least once was common among all people surveyed (NIDA 1993), with nearly one-third stating that they had ever used the drug. Although use was greatest among males ages 26 to 34, use by both males and females ages 18 to 25 was similar, following the pattern described above (table 1).

#### Cocaine Use

Approximately one-tenth of the people surveyed had tried cocaine (NIDA 1993) (table 1). A slightly higher rate of use was seen among adolescent females ages 12 to 17 across all categories. However, because percentages reporting any cocaine use frequency in this age group were small, comparisons require caution. Sampling bias may affect these data, because heavier users of drugs may not live in households and thus may not be captured in a household survey sample (table 1).

#### Heroin Use

Use of heroin was reported by few people surveyed, but household survey results underreport the prevalence of heroin use.^3^ About 1.2 percent of males and 0.6 percent of females had ever used heroin. Use during the past year was greatest for respondents ages 18 to 25, but instead of male and female rates converging in this age group as they did for marijuana, the number of male users was double the number of female users (NIDA 1993).

## Alcohol and Other Drug Abuse Patterns and Consequences

Some differences between patterns and consequences of use by men and women are seen in studies of each of these drugs. Women tend to be younger than men when they begin using alcohol and other drugs and when they first enter treatment (Griffin et al. 1989). Women in alcoholism and other drug abuse treatment list social reasons for their substance abuse more frequently than do men. Specifically, women report use by their male partners as a primary reason for their own substance abuse (Kandel et al. 1986). Typically, more women in treatment also are depressed or suffer from anxiety disorders, whereas more men tend to have antisocial personality disorder^4^ (Griffin et al. 1989; Kosten et al. 1986). The following sections describe additional characteristics of illicit drug abuse by women.

### Studies of Marijuana Use

Findings from studies of marijuana smokers describe behaviors and consequences related to patterns of drug use. A study by Kandel and colleagues (1986) identified marijuana users at ages 15 and 16 and reinterviewed them when they were 24 and 25. The majority of those originally identified still used marijuana. Because most of the heavier smokers (those who had used marijuana at least 1,000 times in their lives) also used alcohol and other drugs frequently, it was difficult to disentangle behaviors related to marijuana use from those related to drinking alcohol, smoking cigarettes, and using other illicit drugs.^5^

Study subjects who had histories of heavy marijuana use had lower rates of marriage and higher rates of abortions among women, divorce or separation among both genders, and higher rates of automobile crashes among men. Job instability, especially unemployment, also correlated with use of marijuana and other drugs by both women and men.

In one series of studies, female marijuana smokers kept daily records of the quantities and times of their alcohol and marijuana use, episodes of sexual activity, mood states, and occurrence of unusual life events (which were defined as “stress”) (Lex et al. 1986; 1989). Diaries collected over a 3-month interval reported the following patterns and effects of marijuana use (Lex et al. 1986):

- On days of marijuana use, light smokers smoked between 0.4 and 1.5 marijuana cigarettes over the 3 months. Heavy smokers smoked between 1.8 and 7.6 marijuana cigarettes over the 3 months.
- Heavy smokers, on average, were 2 to 3 years younger than light smokers when they began to smoke marijuana.
- More marijuana use and more concurrent alcohol and marijuana use occurred on weekends.
- Marijuana use tended to occur earlier in the day on weekdays.
- Heavy smokers reported more daily alcohol use, more days of concurrent alcohol and marijuana use, and a greater frequency of smoking marijuana in the morning.
- Heavy smokers smoked marijuana more frequently when stressful events occurred.

Female heavy and light marijuana users sometimes differed in how they rated their moods (i.e., friendliness, elation, vigor, tension, anger, fatigue, confusion, and depression) associated with their marijuana use (Lex et al. 1989):

- Concurrent use of marijuana and alcohol was associated with increased scores for friendliness and vigor and decreased scores for tension and fatigue for both light and heavy smokers.
- Heavy marijuana smoking influenced almost all mood ratings. Heavy smokers had lower scores for friendliness, elation, and vigor. They had higher scores for tension, anger, fatigue, and confusion but not for depression.
- Sexual activity did not affect negative moods but was associated with increased friendliness, elation, and vigor for both heavy and light smokers.
- Only the mood elation was rated significantly lower on weekdays than on weekends for both light and heavy smokers (however, reports from heavy smokers of lower elation can indicate development of tolerance to marijuana’s euphoric effects).

Further differences in marijuana use between men and women can be seen through laboratory investigations of how environment and access to marijuana affect each gender’s consumption patterns. In two experiments investigating marijuana self-administration in young men (Babor et al. 1974) and women (Babor et al. 1984), participants living in a laboratory in single-gender groups could either exchange points earned by performing a simple task for marijuana cigarettes or could accumulate points until the end of the 35-day study and exchange them for money.

Under these conditions, men increased their marijuana use by one-half or more during the 21 days of the study when marijuana was available (Babor et al. 1974). In sharp contrast, there were no comparable increases for women (Babor et al. 1984). These experiments showed that men’s marijuana smoking appeared to be influenced by availability. It is possible that the laboratory environment, characterized by single-gender groups living together, is more typical of contexts in which men smoke marijuana. Women’s typical marijuana smoking patterns, however, may not occur in groups of women. Instead, women’s use patterns may reflect other social influences, such as the pattern of weekday versus weekend smoking (Lex et al. 1986), influence of male partners (Kandel et al. 1986), or mood states (Lex et al. 1989). For example, female moderate smokers in the laboratory study used more marijuana on days when they reported heightened unpleasant moods, such as anger (Babor et al. 1984), suggesting that increased marijuana smoking could be related to negative affect. Thus, the social environment may play an important role in shaping men’s and women’s patterns of drug use.

### Studies of Cocaine Use

Demographic characteristics of cocaine users differ for men and women, perhaps reflecting disparities in how and when each gender is introduced to the drug. Men in treatment had higher rates of marriage and of employment, especially in a professional, executive, or sales job; and they reported spending more money on cocaine during the past 6 months than did women (Griffin et al. 1989).

Repeating the pattern seen for other drugs, more women in treatment than men lived with a cocaine-dependent partner outside of marriage. Some women also received cocaine from their male partners. Involvement or cohabiting with a drug-dependent partner may have contributed to the more rapid development of cocaine addiction in some women (Griffin et al. 1989).

Women also differed from men in reporting feeling unsociable, experiencing family and job pressures, and having health problems as a consequence of their cocaine use. Compared with women, men reported heightened intoxication effects from cocaine (Griffin et al. 1989).

### Heroin and Other Opiate Use

Hser and colleagues (1990) examined the opiate addiction “careers” of male and female clients in methadone maintenance programs. At admission, approximately 90 percent of the clients had been arrested, and approximately 85 percent were married or had lived with a partner in consensual union and had an average of two to three children.

During the interval between initiation of opiate drug use and physiologic dependence among these clients, women slightly decreased their alcohol use and sharply curtailed nonopiate drug use, such as smoking marijuana. It was conjectured that women may replace use of other drugs with heroin, whereas men continue to experiment simultaneously with many drugs (Hser et al. 1990).

Women took less time than men to become dependent on heroin; many became dependent within 1 month (Hser et al. 1990). Thus, although women and men who use opiates seemed to have similar symptoms of tolerance and dependence, women’s addiction careers were compressed into a shorter cycle. Female opiate users entered treatment after significantly less time, averaging about 5 years from first drug use to admission to a treatment program (versus an average of 8 years for men). This pattern of differential periods of time to dependence for women is consistent with findings from studies of heroin and cocaine addicts. It is reminiscent of “telescoping” of alcohol dependence, whereby women develop physical and social consequences of alcohol abuse faster than do men while consuming the same amount of alcohol over the same period of time.

## Gender Differences of Alcohol and Other Drug Abuse Patterns

In past decades, women’s patterns of alcohol and other drug use (such as those discussed above) were believed to occur only infrequently because women were somehow culturally “protected” by the expectation that they would control their drinking and avoid unrestrained behavior, or the patterns were thought to occur but resemble those of men or to occur only among extremely deviant women (Lex 1991*b*). In more recent years, patterns of substance abuse among women have been examined more closely, but no single explanation has accounted for why women abuse alcohol and other drugs.

A study by Robbins (1989) examined gender differences in psychosocial problems associated with alcohol and other drug abuse by testing three hypotheses. The first hypothesis proposed that variations in women’s substance abuse patterns could be, in part, the result of physiologic differences in the way males and females break down, or metabolize, alcohol and other drugs. Robbins speculated that there may be gender variation in the substances the liver can break down. In support of this hypothesis about physiological gender differences, Frezza and colleagues (1990) demonstrated biological gender differences by showing variations in the initial absorption of alcohol by stomach, or gastric, tissue. In contrast to gastric biopsies from male social drinkers, those from female social drinkers indicated that less activity by alcohol dehydrogenase, the enzyme responsible for metabolizing alcohol, was occurring. Biopsies from female alcoholics revealed almost no gastric alcohol dehydrogenase activity. The difference is important because initial metabolism of alcohol in the stomach diminishes its toxicity. The physiological disparity could result in women having higher levels of alcohol in their bloodstreams and thus remaining intoxicated longer than men; further research on this topic is needed.

Robbins’ second hypothesis proposed that prevailing cultural beliefs hold drug and sometimes alcohol use to be immoral and more stigmatizing for women and that alcohol and other drugs’ potential to compromise women’s sexual chastity or nurturing responsibilities could underlie such disapproval. This idea may be supported by the women in Robbins’ study, who made more efforts than men to hide their substance use and curtailed substance use except when caretaking expectations were in abeyance (Robbins 1989).

The final hypothesis proposed that the different social roles of men and women influence them to express deviant behaviors in different ways. Men’s deviance (in this case, drug abuse) is more public, resulting in aggression and the breaking of rules and laws. Women’s deviance is thought to be channeled into internalized distress (e.g., feeling isolated, distrustful, or helpless) and manifested externally in emotional upset. Robbins’ study demonstrated (in accordance with alcohol and other drug abuse patterns seen in other studies reviewed here) that more women reported depressed and anxious moods.

In Robbins’ study (1989), men used more of both alcohol and other drugs; these data suggested an additional way to explain some gender differences in substance abuse patterns, such as men’s greater frequency of external problems (e.g., legal and financial difficulties).

### Self-Medication in Alcohol and Other Drug Abuse

Another frequently cited theory about the etiology of substance abuse is self-medication, whereby a person’s primary drug of abuse is not selected accidentally but is chosen for its pharmacological ability to relieve specific distressing feelings or symptoms. Many alcohol and other drug-dependent patients claim that they began to use drugs to relieve distress, but few studies support the claim. A study of hospitalized abusers that examined effects of and motivation for alcohol and other drug use (Weiss et al. 1992) showed that use to relieve depressive symptoms was equally common in women with and without major depression but more likely in men with major depression. Thus, a woman with a history of using specific drugs for so-called self-medication may give only limited information about her alcohol and other drug use.

## Adverse Social Consequences of Alcohol and Other Drug Use

### Illicit Drug Use and Crime

Among women, illicit drug use and crime are associated. Approximately one-third of female State prison inmates were under the influence of a drug at the time they committed crimes. One-third also admitted to having used drugs for a month prior to the crime. Drug-related offenses themselves are rising among women. For example, in 1986, 12 percent of all female prisoners were serving a sentence for a drug offense, but by 1991, the proportion was 33 percent (Snell and Morton 1991).

Drug use also appears to influence prostitution and other street crime among women. For example, some women who use crack cocaine exchange sex to obtain it (this is less often true for other drugs). A recent study of prostitution in New York City assessed the impact of crack use (Maher and Curtis 1992). The cost of a cocaine “rock” dropped from $10 to about $2. Concurrent ethnographic fieldwork disclosed that fees for sex acts also decreased, in some instances from $10 or more to $2 to $3 per episode. At the same time, increased numbers of women began to engage in more traditionally male criminal activities, such as assault or robbery. Violent acts by women may reflect decreased income from sexual favors as well as the climate of violence in which prostitution occurs (Maher and Curtis 1992).

### Driving Offenses

Driving under the influence (DUI) offenses have been decreasing for men but increasing among young women (Lex et al. 1994). A recent study examined numerous characteristics of women incarcerated for their third DUI conviction. Of 52 women in this sample, about 70 percent met criteria for illicit drug dependence or abuse in addition to alcohol dependence or abuse (Lex et al. 1994).

### Child Neglect and Abuse

Numerous studies have shown that child neglect and abuse cases are linked commonly to substance use. Some recent estimates point to more than 30,000 infants born to women who use crack cocaine, 10,000 infants born to women who use heroin, and 10 million children being raised by parents who are dependent on alcohol or other drugs. Also, it is believed that at least 675,000 children per year are neglected or abused by such dependent caretakers (Bays 1990). Most women who use illicit drugs are in their childbearing years (18 to 35 years of age), but it is unclear whether alcohol and other drug abuse and child abuse co-occur under comparable family conditions and dynamics or whether substance abuse leads to child abuse. Mediating factors, such as social support, education, income, and parents’ own histories of familial substance abuse and of neglect and abuse, also are important. However, it is likely that when mothers who abuse alcohol and other drugs are primary caregivers, they will neglect some aspect of their children’s emotional or physical needs (Bays 1990).

## Women’s Health Risks Caused by Alcohol and Other Drug Abuse

### Morbidity

Recent reviews (Mello et al. 1989; Mendelson and Mello 1994) provide examples of alcohol and other drug-related medical disorders that challenge the popular belief that alcohol and illicit drugs have only transient and pleasant psychoactive effects. Because all abused drugs have an impact on the female reproductive system (Mello et al. 1989), their effects are likely to be seen in reproductive dysfunction and compromised fertility (table 2).

It generally is reported that women who seek help for obstetric and gynecologic (ob/gyn) problems have a higher rate of substance abuse problems than that of the general female population (Lex 1993). In one study, almost one-third of ob/gyn patients had potential substance abuse problems, and 18 percent reported that infertility or pelvic pain preceded their increased use of alcohol or other mood-altering substances (Busch et al. 1986). Patients reported alleviating pelvic pain with more analgesic drug use, especially during menses.

Abuse of alcohol and other drugs also can compromise other body systems, including the immune system, and complicate existing health problems. For example, intravenous drug use is a major mode of transmission of human immunodeficiency virus (HIV), the virus that causes acquired immunodeficiency syndrome (AIDS). Alcohol abuse can lead to liver and pancreatic diseases and contribute to nutritional deficiencies, which, in their most devastating manifestations, may result in loss of memory and other degenerations of brain function. Marijuana use can worsen psychotic symptoms in schizophrenics and complicate existing heart problems. Cocaine use can cause paranoia and hallucinations, severe depression after withdrawal, fatal respiratory depression, and irregular heart beat. Smoking cocaine delivers the drug to the brain almost immediately, increasing the risk of cerebrovascular accidents (strokes) in the smoker. Chronic cocaine use has been associated with liver damage and with disruption of blood flow in the brain, resulting in dementia (Mendelson and Mello 1994).

### Effects on Pregnancy and the Fetus

There is a range of estimates of the extent to which newborns have been exposed in utero to maternal alcohol or illicit drug use; however NIDA’s National Pregnancy and Health Survey reports that 18.6 percent of infants are exposed to alcohol and 5.4 percent are exposed to any illicit drug in the United States (NIDA 1994). The study also notes that although the women surveyed decreased their use of drugs during pregnancy, they did not discontinue use, a fact that indicates drug addiction’s power over an individual’s ability to control it (NIDA 1994).

Children born to alcohol and other drug-abusing mothers often have lower than normal birth weights, resulting in exorbitant hospital costs for these infants. Drug-exposed infants also are reported to be an average of 1 week less gestational age than nonexposed babies, and almost 20 percent reach a gestational age of less than 37 weeks before birth (Phibbs et al. 1991).

#### Suicide

Women who abuse alcohol and other drugs appear to be at increased risk for attempting suicide. A study by Gomberg (1989) found that almost five times as many alcoholic women (40 percent) as women in the control group (8.8 percent) acknowledged suicide attempts, which occurred even more frequently among the younger alcoholic women. The Drug Abuse Warning Network (DAWN), a voluntary system of reporting drug-related emergency-room visits to NIDA also reports that more than one-half of the drug-related suicide attempts seen in emergency rooms were made by women and that women predominated in drug-related deaths that were designated suicides (NIDA 1992*a*).

#### Emergency Room Data

In 1991 approximately 400,000 patients were treated for drug-related conditions in the 534 hospital emergency services that reported data to DAWN (NIDA 1992*a*). Slightly more than one-half were women. More than one-half the cases involved overdose, and approximately 70 percent of these patients were women. About 0.3 percent of all patients died, with equal proportions among men and women.

Of the more than 685,000 drugs listed in association with these 400,000 emergency room cases, alcohol in combination with other drugs was ranked first in overall frequency, implicated in 36 percent of the men and 26 percent of the women (alcohol data are reported to DAWN only when other drugs also are involved). Cocaine ranked second in 35 percent of the men and 17 percent of the women. Narcotic analgesics, including heroin or morphine, methadone, codeine combinations, and other opioids, ranked third in overall frequency.

#### Medical Examiner Cases

Data from cases reported by 130 medical examiners in departments across the country reveal that almost three-quarters of the reported deaths involved multiple drug use (NIDA 1992*b*). These drug use patterns were comparable for men and women. Alcohol in combination with other drugs was associated with slightly more than one-third of the deaths. Narcotic analgesics, including heroin, methadone, codeine, and propoxyphene (Darvon^®^), were associated with more than one-half of the deaths. Cocaine was associated with just under one-half of the deaths (overlap exists in these groupings, indicating multiple substance use). Rankings for the number of deaths caused by these drugs were identical for each gender, but greater proportions of each category were men (NIDA 1992*b*).

## Summary

As demonstrated by experimental, clinical, and survey findings, the consequences of alcohol and other drug abuse by women are serious and sometimes life threatening. Suicide attempts are more common among women, but more so among young women who abuse alcohol along with other drugs than among women who do not abuse alcohol and other drugs. Women now have increased rates of incarceration for drug-related offenses, more drug-related cases of child neglect or abuse, greater drug-related involvement with prostitution, and increased rates of driving under the influence of alcohol. Substance abuse among women also has been associated with job instability, especially unemployment; lower rates of marriage; and higher rates of abortion and divorce.

Women and men appear to differ in their motivation to use alcohol and other drugs and to experience different consequences of drug use. Factors contributing to these trends are complex, involving male-female relationships and the drugs’ effects on the psychological states of the users.

To identify women with substance abuse problems and begin interventions at the earliest opportunity, the context in which a woman first tries an illicit drug and the circumstances that permit or promote continued and sustained drug use must be studied. Under the best circumstances, such studies would inquire about the use of alcohol along with other drugs, so that the most accurate picture of multiple substance use among women would emerge.

## Figures and Tables

**Table 1:** 1992 Prevalence Estimates for Use of Alcohol, Illicit Drugs, Marijuana, and Cocaine: Ever, Past Year, and Past Month by Age and Gender Groups for Total U.S. Population^1^

Age (years)	Gender	Ever Used (%)	Used in Past Year (%)	Used in Past Month (%)
**Alcohol Use**				

12–17	Combined	39.3	32.6	15.7
	Male	41.6	33.7	16.9
	*Female*	*37.0*	*31.4*	*14.5*
18–25	Combined	86.3	77.7	59.2
	Male	87.7	79.8	65.6
	*Female*	*85.0*	*75.6*	*53.0*
26–34	Combined	91.7	79.0	61.2
	Male	93.4	83.6	70.0
	*Female*	*90.0*	*74.5*	*52.8*
35+	Combined	87.0	62.6	46.5
	Male	93.8	69.0	56.1
	*Female*	*81.0*	*57.0*	*38.0*
Total	Combined	83.0	64.7	47.8
	Male	87.3	69.5	55.9
	*Female*	*79.0*	*60.2*	*40.4*

**Illicit Drug Use**				

12–17	Combined	16.5	11.7	6.1
	Male	16.3	11.0	5.7
	*Female*	*16.6*	*12.5*	*6.5*
				
18–25	Combined	51.7	26.4	13.0
	Male	53.3	30.4	16.7
	*Female*	*50.0*	*22.6*	*9.5*
				
26–34	Combined	60.8	18.3	10.1
	Male	66.1	22.3	12.6
	*Female*	*55.5*	*14.4*	*7.6*
				
35+	Combined	28.0	5.1	2.2
	Male	34.1	6.7	3.2
	*Female*	*22.8*	*3.7*	*1.4*
				
Total	Combined	36.2	11.1	5.5
	Male	41.0	13.4	7.1
	*Female*	*31.7*	*9.0*	*4.1*

**Marijuana Use**				

12–17	Combined	10.6	8.1	4.0
	Male	11.6	8.7	4.6
	*Female*	*9.6*	*7.5*	*3.5*
18–25	Combined	48.1	22.7	11.0
	Male	49.7	27.1	14.5
	*Female*	*46.6*	*18.4*	*7.5*
26–34	Combined	58.6	14.3	8.2
	Male	64.3	18.9	11.0
	*Female*	*52.9*	*9.9*	*5.5*
35+	Combined	24.8	3.3	1.6
	Male	31.2	4.5	2.3
	*Female*	*19.2*	*2.2*	*1.0*
Total	Combined	32.8	8.5	4.4
	Male	38.0	10.8	5.9
	*Female*	*28.0*	*6.3*	*2.9*

**Cocaine Use**				

12–17	Combined	1.7	1.1	0.3
	Male	1.6	1.0	0.2
	*Female*	*1.8*	*1.2*	*0.3*
18–25	Combined	15.8	6.3	1.8
	Male	18.8	8.5	2.9
	*Female*	*12.9*	*4.2*	*0.8*
26–34	Combined	25.2	4.9	1.4
	Male	29.8	6.3	1.7
	*Female*	*20.8*	*3.5*	*1.1*
35+	Combined	6.9	0.9	0.2
	Male	8.8	1.3	0.3
	*Female*	*5.2*	*0.6*	*0.1*
Total	Combined	11.0	2.4	0.6
	Male	13.4	3.2	0.9
	*Female*	*8.7*	*1.7*	*0.4*

1 U.S. population estimate = 205,713,288 people.

SOURCE: National Institute on Drug Abuse 1993.

**Table 2:** Derangements of Reproductive Function Reported for Women Who Used Cocaine, Opiates, or Marijuana

	Cocaine	Opiates	Marijuana
Amenorrhea^1^	×	×	×
Anovulation^2^	×	×	×
Luteal Phase Dysfunction^3^	×	×	×
Hyperprolactinemia^4^	×	×	
Spontaneous Abortion^5^	×	×	×

1 Absence or abnormal cessation of menses.

2 Absence of ovulation.

3 Disorder of the luteal phase of the menstrual cycle seen as either a short luteal phase defect (8 days or less from ovulation to menses) or an inadequate luteal phase (when progesterone levels are abnormally low) that can prevent pregnancy.

4 Increased levels of prolactin, a hormone that stimulates lactation in mammals, in the blood. In women, this condition may cause amenorrhea or disruptions of menstrual cycle regularity.

5 Abortion of a fetus that occurs naturally.

SOURCE: Mello et al. 1989.

## Footnotes

1 In this article, these terms will be defined as they are in each of the studies discussed.

2 “Sampling error” refers to possible differences between results that could have been obtained from a complete survey of all the people in a population and the results that do occur if only randomly selected portions of the population are surveyed. In this data set, sampling error averaged 3 percent.

3 Once information from the 1993 Household Survey becomes available, a clearer picture of heroin use in the household population can emerge. The survey is planned to increase overall sample size by pooling heroin use data from the 1991, 1992, and 1993 surveys to gain more information about the frequency of use by age, gender, and ethnicity of users (SAMHSA 1993).

4 Antisocial personality disorder as defined by the American Psychiatric Association’s *Diagnostic and Statistical Manual of Mental Disorders, Third Edition, Revised* (1987) includes “a pattern of irresponsible and antisocial behavior since the age of 15,” and is indicated by any combination of a set of recognized symptoms (pp. 342–346).

5 Among these users, 37 percent of males and 24 percent of females also reported using cocaine, 32 percent of males and 19 percent of females had used psychedelics, 30 percent of males and 19 percent of females had used stimulants, and 20 percent of males and 15 percent of females had used benzodiazepines or other minor tranquilizers without prescription.

## References

[b1-arhw-18-3-212] American Psychiatric Association (1987). Diagnostic and Statistical Manual of Mental Disorders, Third Edition, Revised.

[b2-arhw-18-3-212] Babor TF, Mendelson JH, Greenberg I, Kuehnle JC (1974). Marihuana consumption and tolerance to physiological and subjective effects. Archives of General Psychiatry.

[b3-arhw-18-3-212] Babor TF, Lex BW, Mendelson JH, Mello NK, Harris LS (1984). Marijuana, effect and tolerance: A study of subchronic self-administration in women. Problems of Drug Dependence, 1983.

[b4-arhw-18-3-212] Bays J (1990). Substance abuse and child abuse: Impact of addiction on the child. Pediatric Clinics of North America.

[b5-arhw-18-3-212] Busch D, McBride AB, Benaventura LM (1986). Chemical dependency in women: The link to ob/gyn problems. Journal of Psychosocial Nursing and Mental Health Services.

[b6-arhw-18-3-212] Ferrence RG, Whitehead PC, Kalant OJ (1980). Sex differences in psychoactive drug use: Recent epidemiology. Research Advances in Alcohol and Drug Problems. Volume 5: Alcohol and Drug Problems in Women.

[b7-arhw-18-3-212] Frezza M, di Padova C, Pozzato G, Terpin M, Baragna E, Lieber CS (1990). High blood alcohol levels in women: The role of decreased gastric alcohol dehydrogenase activity and first-pass metabolism. New England Journal of Medicine.

[b8-arhw-18-3-212] Gomberg ESL (1989). Alcoholism in women: Use of other drugs. Alcoholism: Clinical and Experimental Research.

[b9-arhw-18-3-212] Griffin ML, Weiss RD, Mirin SM, Lange U (1989). A comparison of male and female cocaine abusers. Archives of General Psychiatry.

[b10-arhw-18-3-212] Hser YI, Anglin MD, Powers K, Galanter M (1990). Longitudinal patterns of alcohol use by narcotics addicts. Recent Developments in Alcoholism. Volume 8. Combined Alcohol and Other Drug Dependence.

[b11-arhw-18-3-212] Kandel DB, Davies M, Karus D, Yamaguchi K (1986). The consequences in young adulthood of adolescent drug involvement. Archives of General Psychiatry.

[b12-arhw-18-3-212] Kosten TR, Rounsaville BJ, Kleber HD (1986). Ethnic and gender differences among opiate addicts. International Journal of Addictions.

[b13-arhw-18-3-212] Lex BW (1987). Review of alcohol problems in ethnic minority groups. Journal of Consulting and Clinical Psychology.

[b14-arhw-18-3-212] Lex BW, Watson RR (1991a). Prevention of substance abuse problems in women. Alcohol and Drug Abuse Reviews.

[b15-arhw-18-3-212] Lex BW (1991b). Some gender differences in alcohol and polysubstance users. Health Psychology.

[b16-arhw-18-3-212] Lex BW, Gomberg ESL, Nirenberg T (1993). Women and illicit drugs: Marijuana, heroin, and cocaine. Women and Substance Abuse.

[b17-arhw-18-3-212] Lex BW, Griffin ML, Mello NK, Mendelson JH (1986). Concordant alcohol and marihuana use in women. Alcohol.

[b18-arhw-18-3-212] Lex BW, Griffin ML, Mello NK, Mendelson JH (1989). Alcohol, marijuana, and mood states in young women. International Journal of the Addictions.

[b19-arhw-18-3-212] Lex BW, Goldberg ME, Mendelson JH, Lawler NS, Bower T (1994). Components of antisocial personality disorder among women convicted for drunken driving. Annals of the New York Academy of Sciences.

[b20-arhw-18-3-212] Maher L, Curtis R (1992). Women on the edge of crime: Crack cocaine and the changing contexts of street level sex work in New York City. Crime, Law, and Social Change.

[b21-arhw-18-3-212] Mello NK, Mello NK (1983). Etiological theories of alcoholism. Advances in Substance Abuse: Behavioral and Biological Research.

[b22-arhw-18-3-212] Mello NK, Mendelson JH, Teoh SK (1989). Neuroendocrine consequences of alcohol abuse in women. Annals of the New York Academy of Sciences.

[b23-arhw-18-3-212] Mendelson JH, Mello NK, Isselbacher KJ, Braunwald E, Wilson JD, Martin JB, Fauci AS, Kasper DL (1994). Cocaine and other commonly abused drugs. Harrison’s Principles of Internal Medicine.

[b24-arhw-18-3-212] National Institute on Drug Abuse (1992a). Annual Emergency Room Data, 1991.

[b25-arhw-18-3-212] National Institute on Drug Abuse (1992b). Annual Medical Examiner Data, 1991.

[b26-arhw-18-3-212] National Institute on Drug Abuse (1993). National Household Survey on Drug Abuse: Population Estimates 1992.

[b27-arhw-18-3-212] National Institute on Drug Abuse National Pregnancy and Health Survey: Initial Findings.

[b28-arhw-18-3-212] Phibbs CS, Bateman DA, Schwartz RM (1991). The neonatal costs of maternal cocaine use. Journal of the American Medical Association.

[b29-arhw-18-3-212] Robbins C (1989). Sex differences in psychosocial consequences of alcohol and drug abuse. Journal of Health and Social Behavior.

[b30-arhw-18-3-212] Snell TL, Morton DC (1991). Women in Prison: Survey of State Prison Inmates, 1991.

[b31-arhw-18-3-212] Substance Abuse and Mental Health Services Administration (1993). National Drug and Alcoholism Treatment Unit Survey (NDATUS): 1991 Main Findings Report.

[b32-arhw-18-3-212] Weiss RD, Griffin ML, Mirin SM (1992). Drug abuse as self-medication for depression: An empirical study. American Journal of Drug and Alcohol Abuse.

